# Novel modified endoscopic mucosal resection of large GI lesions (> 20 mm) using an external additional working channel (AWC) may improve R0 resection rate: initial clinical experience

**DOI:** 10.1186/s12876-020-01344-6

**Published:** 2020-06-19

**Authors:** A. Sportes, Jung CFM, M. A. Gromski, P. Koehler, A. Seif Amir Hosseini, P. Kauffmann, V. Ellenrieder, E. Wedi

**Affiliations:** 1 Department of Gastroenterology, Institut Arnault Tzanck, Saint Laurent du Var, France; 2 Department of Gastroenterology and Gastrointestinal Oncology, Interdisciplinary Endoscopy, University Medical Center, Georg-August-University, 37075 Goettingen, Germany; 3 Division of Gastroenterology and Hepatology, Indiana University School of Medicine, Indianapolis, IN USA; 4 Institute of Farm Animal Genetics, Friedrich-Loeffler-Institut (FLI), Federal Research Institute for Animal Health, Mariensee, Germany; 5 Department of Diagnostic and Interventional Radiology, University Medical Center, Göttingen, Germany; 6 Department of Oral and Maxillofacial Surgery, University Medical Center Goettingen, Göttingen, Germany

**Keywords:** Endoscopic mucosal resection (EMR), Endoscopic mucosal resection plus (EMR+) technique, Additional working channel (AWC), Triangulation

## Abstract

**Background:**

En-bloc resection of large, flat dysplastic mucosal lesions of the luminal GI tract can be challenging. In order to improve the efficacy of resection for lesions ≥2 cm and to optimize R0 resection rates of lesions suspected of harboring high-grade dysplasia or early adenocarcinoma, a novel grasp and snare EMR technique utilizing a novel over the scope additional accessory channel, termed EMR Plus (EMR+), was developed. The aim of this pilot study is to describe the early safety and efficacy data from the first in human clinical cases.

**Methods:**

A novel external over-the-scope additional working channel (AWC) (Ovesco, Tuebingen, Germany) was utilized for the EMR+ procedure, allowing a second endoscopic device to be used through the AWC while using otherwise standard endoscopic equipment. The EMR+ technique allows tissue retraction and a degree of triangulation during endoscopic resection. We performed EMR+ procedure in 6 patients between 02/2018–12/2018 for lesions in the upper and lower GI tract.

**Results:**

The EMR+ technique utilizing the AWC was performed successfully in 6 resection procedures of the upper and/or lower GI tract in 6 patients in 2 endoscopy centers. All resections were performed successfully with the EMR+ technique, all achieving an R0 resection. No severe adverse events occurred in any of the procedures.

**Conclusions:**

The EMR+ technique, utilizing an additional working channel, had an acceptable safety and efficacy profile in this preliminary study demonstrating it’s first use in humans. This technique may allow an additional option to providers to remove complex, large mucosal-based lesions in the GI tract using standard endoscopic equipment and a novel AWC device.

## Background

Endoscopic en-bloc resection of lateral-spreading adenomas, flat lesions larger > 2 cm and/or early cancer of the luminal GI tract can be challenging, even for the experienced endoscopist. Endoscopic mucosal resection (EMR) is widely performed and is an effective, minimally invasive endoscopic strategy for patients with large neoplastic mucosal based lesions. In the colon, saline-assisted snare resection of flat lesions (classic EMR) can be considered the established “gold-standard” technique [1, 2].

In the esophagus, the most commonly employed EMR techniques are Cap-EMR and the band and snare technique [1]. For gastric lesions, cap resection for lesions up to 1 cm is feasible. For larger lesions, saline-assisted snare resection of flat lesions (classic “EMR”) is proposed, similar to the duodenum as well. Piece-meal resection is usually used for large lesions (> 2 cm) and is effective, but often R0 resection cannot be assessed by the pathologist. This distinction is not necessarily important in the case of low-grade dysplasia, but in the case of high-grade dysplasia or early adenocarcinoma this plays a crucial role [1]. For high-risk lesions Endoscopic submucosal dissection (ESD) is an option with a better chance of R0 resection for larger lesions. But ESD is an advanced expert technique with a long learning curve, has a longer procedure time than EMR and a higher incidence of adverse events. One of the major complications is perforations, with an incidence up to 4 – 10% [2, 3].

During complex EMR, generally a single channel endoscope is used, with the channel being utilized by the snare. There have been prior publications describing the resection of large mucosal-based lesions with a modified EMR technique that allows for traction of the lesion wherein a double channel upper endoscope was used [4]. The double channel therapeutic endoscope may not be readily available in community practices. Furthermore, this scope can make it difficult to gain access to difficult to reach lesions (e.g., right colon and duodenal sweep). Another disadvantage of a double channel scope is that the small and fixed distance between the two working channels minimizes triangulation options.

For these reasons, we believe that the classical EMR technique may be enhanced by a new external additional working channel (AWC, Ovesco Endoscopy, Tuebingen, Germany), to allow for traction and countertraction of the mucosal lesion as well as to allow a larger resection area, termed the “EMR+ technique”. This case series aims to evaluate this novel tool and approach in a clinical setting. #.

## Methods

**Fig. 1:**
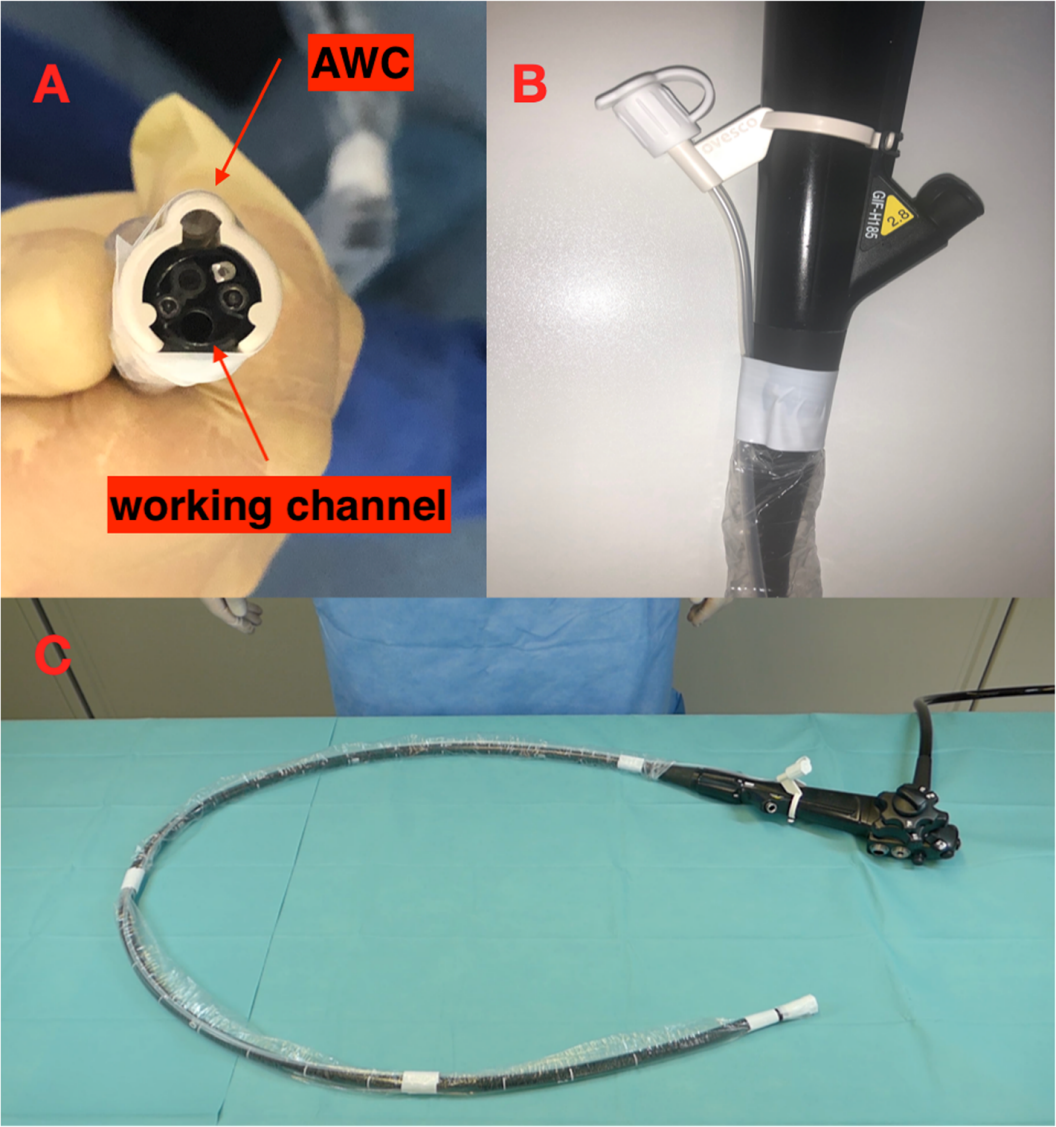
**a** AWC mounted on the tip of the endoscope with a freely adjustable distance to the regular working channel. **b** AWC-valve attached to the shaft of the endoscope. **c** External installation of the AWC on a single-channel gastroscope

**Fig. 2:**
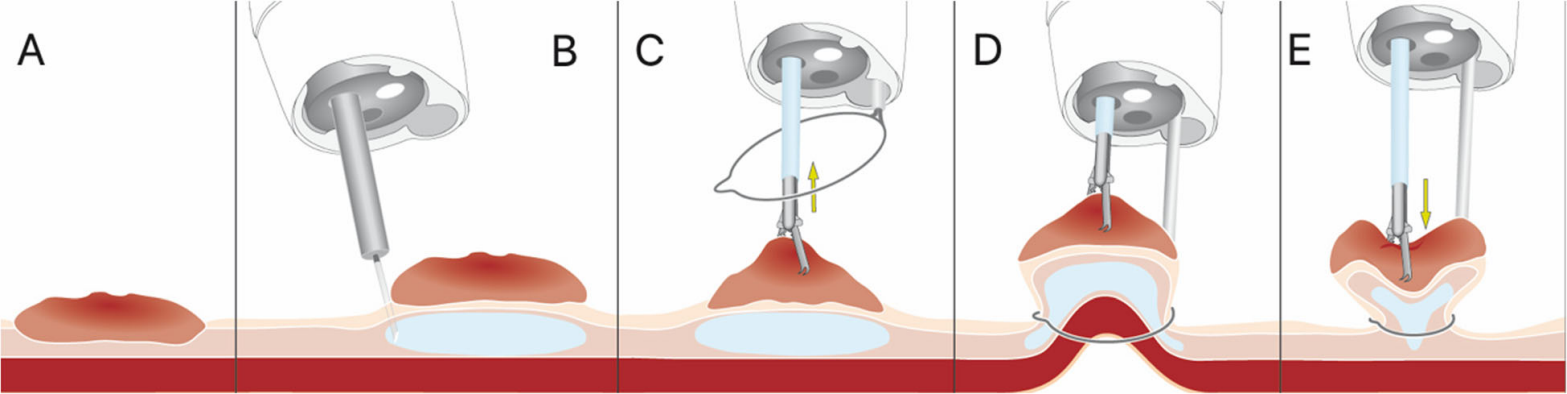
Step by step description of endoscopic mucosal resection with an additional working channel (EMR+ technique) (AWC, Ovesco Endoscopy AG, Tuebingen, Germany). **a** Target lesion, **b** Submucosal injection (e.g. HAES 0.6) for elevation of the lesion. **c** Grasping of the lesion through a 25 mm snare. **d** Retraction of the lesion with the grasper and closure of the snare. **e** Forwards pushing (pushing-back) of the grasper through the closed snare (to free any entrapped muscularis) followed by endoscopic resection

The AWC utilized for the EMR+ procedures consists of a flexible endoscopic-tip attachment, which can be used with standard-issue upper and lower video endoscopes. The AWC has a shaft with a length of either 122 cm (for endoscopes with insertion lengths: 103–110 cm) or 185 cm (for endoscopes with insertion lengths: 160–170 cm), an adaptor for fixation at the endoscope handle with Luer-lock attachment, a valve and a sleeve with adhesion tape (Fig. 1). The AWC allows passage of instruments with an outer diameter of up to 2.8 mm (7Fr), and may be utilized with endoscopes with tip diameters ranging from 8.5 to 13.5 mm. The additional working channel is able to be rotated manually up to 360 degrees on the distal tip of the endoscope, allowing for variable position of tool presentation at the scope tip. This enables the user to modify the distance between the channels, which helps to improve the maneuverability of the instruments. A step-by-step description of the EMR+ procedure is illustrated in Fig. 2.

This study was a pilot-study performed at the University Hospital of Goettingen, Germany and Institut Arnault Tzanck, Saint Laurent du Var, France from January 2018 to February 2019. The research study was approved by the institutional review board (IRB) of both institutions and a written consent was given from all patients. Inclusion criteria were: age ≥ 18 years, neoplastic mucosal-based lesions in the stomach or colon with a size of ≥2 cm, an indication for endoscopic removal and without lymphonodal involvement on prior abdominal ultrasound. Exclusion criteria were: patients physical unfit for endoscopic resection, suspicion for invasive malignancy or lymphonodal involvement, indication for surgical resection and anatomical changes preventing endoscopic access. Descriptive analysis was performed on the data. All patients had prior endoscopy to resection. Technical and clinical success were defined as complete resection (en-bloc/R0) of lesion defined by gross inspection by endoscopist and routine histopathological analysis. Adverse events were divided into major complications such as death, bleeding and perforation and minor complications such as additional mucosal damage by the AWR/endoscope. Routine short-term follow-up endoscopy was not part of this study.

All interventions were performed using Olympus GIF-1TH190 or Olympus GIF-HQ190 endoscopes by a single experienced interventional endoscopist. In the reported cases, the endoscopist worked with one experienced endoscopic nurse handling the tools for AWC and the endoscopist used the tool in the standard working channel.

All procedures were performed with the novel AWC device using the previously described EMR+ technique (Fig. 2). After submucosal injection of HAES 6% (B. Braun, Melsungen, Germany), the mucosal-based lesions were successfully resected (Endocut Q 1/1/1, ERBE VIAO 200, ERBE Elektromedizin, Tübingen, Germany) with a goal of en-bloc capture, with a 25 mm snare (Captivator II, Boston Scientific) after creating traction of the mucosal lesion using a standard grasper via the additional working channel (AWC) through the snare (Fig. 2 C-D). During pre-clinical experiments on the EMR+ technique using a porcine ex-vivo model [3], we found that pushing back the grasped tissue gently immediately prior to resection appeared to reduce the risk of muscular involvement or perforation (Fig. 2 E).

## Results

**Fig. 3:**
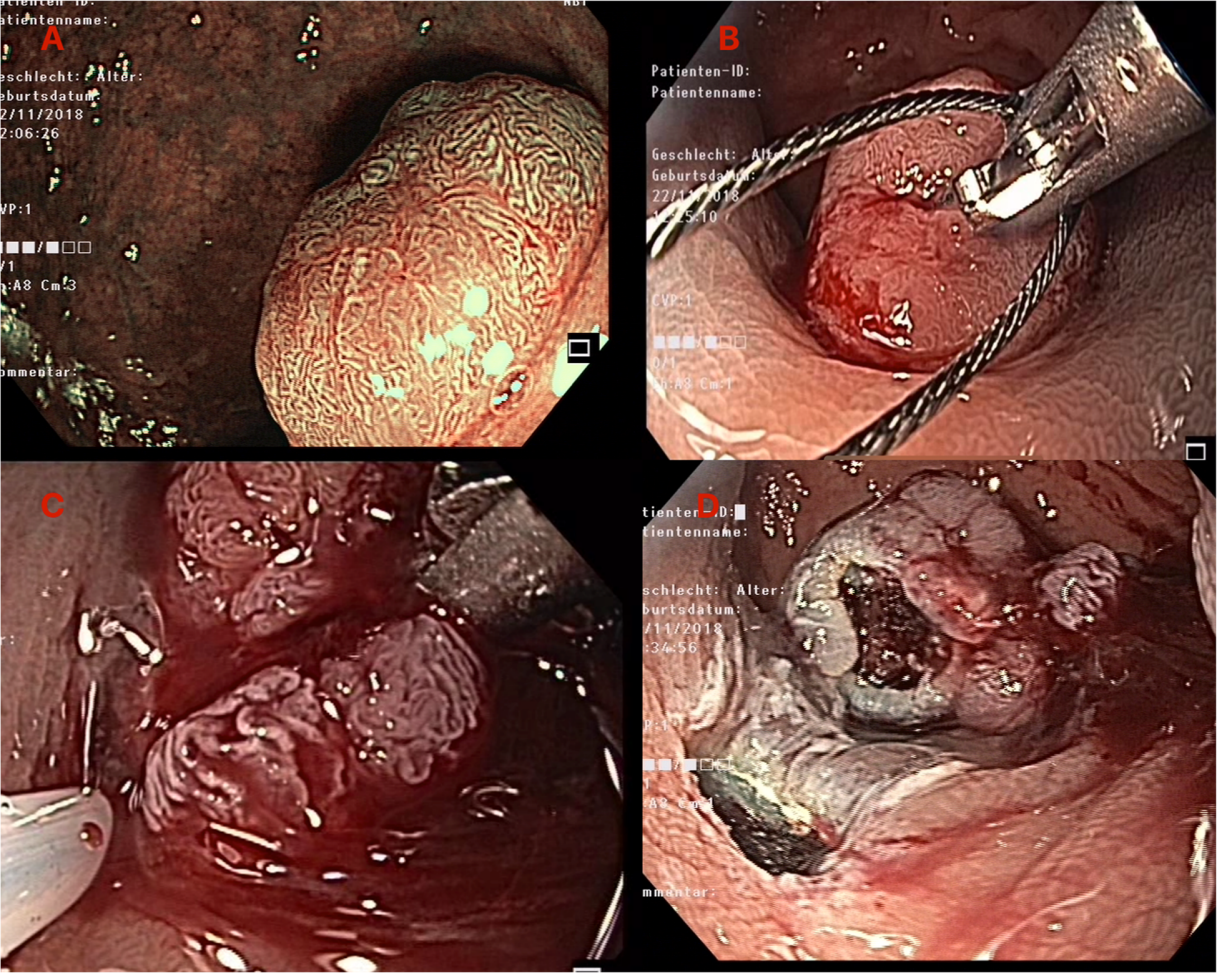
Clinical case of EMR+ procedure. **a** Target lesion, Paris 0-Ip, size 30 mm. **b** After submucosal injection of HAES 0.6% and mounting of the AWC on the scope the lesion is grasped through a 25 mm snare. **c** Retraction of the lesion with the grasper and closure of the snare. **d** Followed by endoscopic resection. Histopathological examination revealed tubulovillous adenoma with low-grade dyplasia, R0 resection

Resection of mucosal-based GI lesions was attempted in 6 patients, with the EMR+ technique using the AWC (2 upper gastrointestinal tract, 4 lower gastrointestinal tract). All resections were successful for an en-bloc resection of the lesion, confirmed by histopathological analysis. A representative example of a resection is demonstrated in Fig. 3.

The average estimated lesion size was 30.8 mm (median 30,83 mm). The median age of the patients was 76 years (3 female, 3 male). Mean procedure time was 25.5 min. In 2 cases, there was intra-procedural bleeding, which was managed endoscopically via a hemoclip application. There were no post-interventional adverse events (perforation/bleeding).

In 4 cases histological examination revealed tubulovillous adenoma with low-grade dysplasia, one case was a tubulovillous adenoma with focal high-grade dysplasia and one case was a sessile serrated adenoma with low-grade dysplasia. In all cases, R0-resection was achieved (Table 1). During a follow-up of 6 months no further endoscopic or surgical treatment was subsequently needed.

**Table 1:**
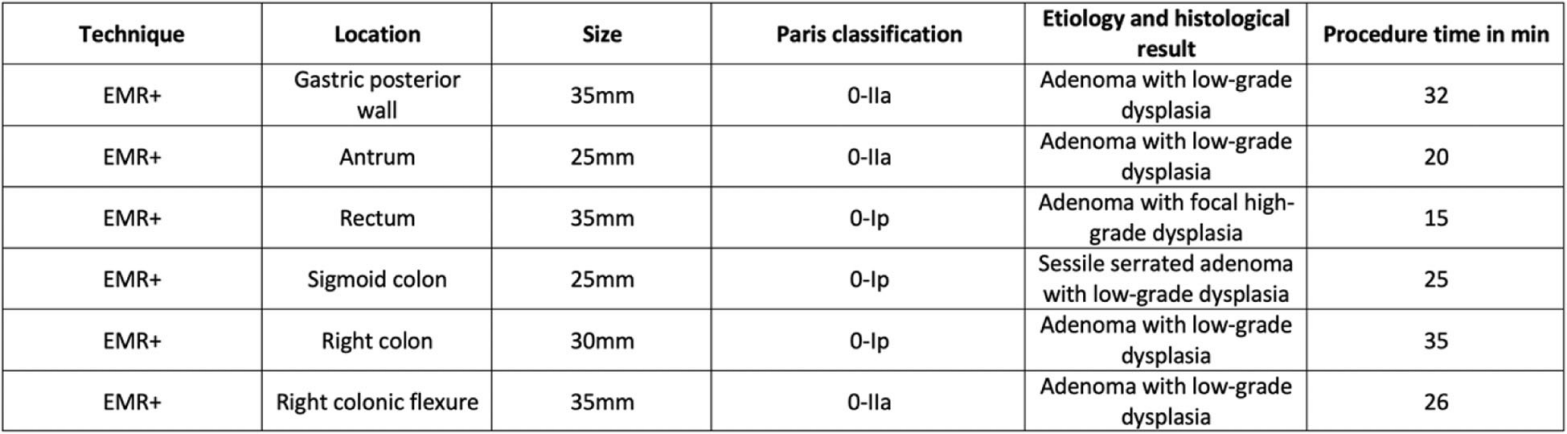
Overview of the procedural details

## Discussion

Resection techniques for non-invasive neoplasia of the GI mucosa have evolved from conventional endoscopic mucosal resection (EMR) to endoscopic submucosal dissection (ESD) and endoscopic full-thickness resection (EFTR) [5]. EMR and ESD are established techniques in interventional endoscopy for the treatment of dysplastic and select early malignant lesions. But, EMR bears certain limitations in the treatment of flat lesions sized ≥2 cm. In these cases, piece-meal EMR often is required for complete resection, which may hinder complete pathological evaluation and also may affect the recurrence risk of certain lesions [1, 6].

The modified grasp and snare EMR technique (EMR+) with the use of an additional external working channel (AWC) may offer an alternative to available techniques. The AWC allows the introduction of an additional grasping tool (e.g. grasping forceps or anchor) for traction or counter-traction. Another advantage is that the positioning of the AWC can be customized to the clinical scenario, depending on the position of the lesion and optimal angle of exit for the second tool [7].

In a recently published case series Walter et al. describe that a larger distance between the two working channels could enable the endoscopist to make better use of the traction and counter-traction principle and enable more effective use of leverage effect [8]. They performed 4 EMR+ procedures > 30 mm and had an R0 resection rate of 50%. Two lesions > 45 mm had to be resected in piece-meal technique. They used a 40 mm snare for the EMR+ procedure [8]. In comparison, we used a 25 mm snare and could achieve an en-bloc resection rate of 100% in lesion sizes up to 35 mm. We believe that further studies are required to determine the optimal snare and traction tools to be used. After preliminary experiments in porcine stomachs, we learned that after retraction of the lesion into the snare and snare closure, a push-back maneuver to release pressure on the muscularis is helpful to prevent perforations.

Like any technique, the EMR+ does have certain drawbacks. Due to the external fixation outside the scope of the AWC, the diameter of the entire scope tip increases to an additional 3 mm, and this can make passage of tight anatomic locations (e.g., pharynx, terminal ileum, etc.) more difficult [8]. Additionally, EMR+ procedures in the right hemi colon or cecum can be challenging with the AWC due to a long distance to pass through the colon. Care should be also taken due to the stiff external scope tip, in case of incautious advancement, it may damage the mucosae or the muscularis mucosae. Furthermore, although the “push-back” maneuver has improved the degree of muscular injury or perforation (with no perforations in this series), there remains potential for damage to the muscularis propria with the traction maneuver followed by resection, which should be cautioned for very large flat lesions (> 35 mm).

In general, comparing EMR+ with AWC to standard EMR procedures, prospective, randomized studies in human are not available. From our own experience, R0 en bloc resection with the EMR+ method was feasible even in lesions up to 35 mm. R0 resection rates by standard EMR for this size are much lower and according to us, can be improved by the AWC technique in a porcine model [9]. Our study group recently showed in a porcine model, that with the grasp-and-snare technique, EMR+ facilitates en bloc resection of larger lesions compared to conventional EMR. In lesions 2 cm and larger, EMR+ has demonstrated advantages, especially concerning en bloc resection rate. It seems that at 3 cm, EMR+ reaches its best discriminatory power whereas EMR+ has inherent limits at 4 cm and in lesions of that size, other techniques such as ESD or surgery should be considered [9].

A serious advantage is the traction / countertraction manoeuvrability, which is offered by the AWC. Therefore, clear and safe snare positioning is possible when compared to standard EMR. With the mounted AWC, the endoscope becomes more rigid and maneuvers are a bit less comfortable to undertake.

In this case series, no routine second-look endoscopy was performed in order to evaluate for mucosal damage after initial resection. All patients in our series showed no clinical or laboratory signs to indicate emergency endoscopy, so no follow-up was performed. The extent of mucosal alterations after resection with the AWC where of normal size and quality, comparable to normal EMR.

There is a need for larger studies to validate the effectiveness of this new technique. Future study designs may also incorporate second-look endoscopies for screening of mucosal damage after initial resection.

## Conclusions

We conclude that based on our preliminary experience, the newly developed external AWC may be a useful tool in an EMR+ procedure to help resect larger lesions in the upper or lower gastrointestinal tract safely and efficiently. We hope to pursue further studies of this procedure, and eventually a comparison of EMR+ to ESD for safety and efficiency of R0 resection of neoplastic mucosal lesions in the luminal GI tract would be of interest.

## Data Availability

The datasets used and/or analyzed during the current study are available from the corresponding author on reasonable request.

## Abbreviations

AWC: Additional working channel
EMR: Endoscopic mucosal resection
ESD: Endoscopic submucosal dissection
GI: Gastrointestinal
HAES: Hydroxyethyl starch

## References

[1] Hochberger J, Kruse E, Wedi E, Buerrig KF, Dammer S, Koehler P, Cohen J (2011). Training in endoscopic mucosal resection and endoscopic submucosal dissection. Successful gastrointestinal endoscopy.

[2] Hochberger J, Kohler P, Kruse E, Huppertz J, Delvaux M, Gay G (2013). Endoscopic submucosal dissection. Internist.

[3] Wedi E, Koehler P, Hochberger J, Maiss J, Milenovic S, Gromski M (2019). Endoscopic submucosal dissection with a novel high viscosity injection solution (LiftUp) in an ex vivo model: a prospective randomized study. EndoscopyI Int Open.

[4] Jung Y, Kato M, Lee J, Gromski MA, Chuttani R, Matthes K (2013). Effectiveness of circumferential endoscopic mucosal resection with a novel tissue-anchoring device. World J Gastrointest Endosc.

[5] Wedi E, Orlandini B, Gromski M, Jung CFM, Tchoumak I, Boucher S (2018). Full-thickness resection device for complex colorectal lesions in high-risk patients as a last-resort endoscopic treatment: initial clinical experience and review of the current literature. Clin Endosc.

[6] Holmes I, Kim HG, Yang DH, Friedland S (2016). Avulsion is superior to argon plasma coagulation for treatment of visible residual neoplasia during EMR of colorectal polyps (with videos). Gastrointest Endosc.

[7] Wedi E, Knoop RF, Jung C, Ellenrieder V, Kunsch S (2019). Use of an additional working channel for endoscopic mucosal resection (EMR +)of a pedunculated sessile serrated adenoma in the sigmoid colon. Endoscopy.

[8] Walter B, Schmidbaur S, Krieger Y, Meining A (2019). Improved endoscopic resection of large flat lesions and early cancers using an external additional working channel (AWC): a case series. EndoscopyI Int Open.

[9] Knoop RF, Wedi E, Petzold G, Bremer SCB, Amanzada A, Ellenrieder V (2020). Endoscopic mucosal resection with an additional working channel (EMR+) in a porcine ex vivo model: a novel technique to improve en bloc resection rate of snare polypectomy. EndoscopyI Int Open.

